# Endolymphatic sac tumor: case report and review of the literature

**DOI:** 10.1186/1746-1596-7-36

**Published:** 2012-04-02

**Authors:** Yan-Hua Sun, Wen Wen, Jun-Hui Wu, Jian-Ming Song, Hong Guan, Kai-Xin Wang, Mei-Quan Xu

**Affiliations:** 1 Department of Pathology, Shenzhen Second People's Hospital, 3002 Sungang West Road, Shenzhen 518035, China; 2 Department of Pathology, Shenzhen Nanshan District People's Hospital, 89 TaoYuan Road, Shenzhen 518067, China

**Keywords:** Endolymphatic sac tumor, ELST, Heffner tumor, Aggressive papillary middle ear tumor, von Hippel-Lindau disease

## Abstract

Endolymphatic sac tumor (ELST) is a rare neoplasm which can be encountered sporadically or in Von Hippel-Lindau (VHL) disease. Here we report a sporadic case of ELST in 31-year-old man. Neither the symptoms nor a family history of VHL disease were found in the patient. CT imaging demonstrated an expansile lytic lesion of the mastoid process of the left petrous bone. MR scanning revealed a 5.2 cm × 4.7 cm × 4.2 cm mass which showed hyperintensity on T1- and T2-weighted images. Histologic sections showed a papillary, cystic or glandular architecture. The papillary and glandular structures were lined by a single layer of flattened cuboidal-to-columnar cells. The stroma of the papillary fronds was richly vascularized and chronically inflamed. The tumor showed diffusely positive reactivity with cytokeratin (Pan), cytokeratin 19, cytokeratin 5/6, cytokeratin 7, EMA, vimentin, CD56, and NSE and also showed variable reactivity with glial fibrillary acidic protein (GFAP) and VEGF. The Ki-67 immunostain showed a proliferation index of < 1%. Because the mass was large, it was difficult to extirpate surgically. After surgery, the patient underwent gamma-knife radiosurgery for residual tumor. The findings indicate that ELST is a rare neoplasm with benign histopathological appearance and clinically destructive behavior. Because of the rarity of this tumor, it can easily be confused with other tumors such as paraganglioma, middle ear adenoma, adenocarcinoma, papillary carcinoma of thyroid or choroid plexus papilloma. Owing to its locally aggressive nature, it is difficult to extirpate surgically when it is large.

**Virtual Slides:**

The virtual slide(s) for this article can be found here: http://www.diagnosticpathology.diagnomx.eu/vs/7973320646763012

## Background

Endolymphatic sac tumor (ELST) is a rare neoplasm with benign histopathological appearance and clinically destructive behavior which occurs in the skull base and frequently invades the posterior petrous bone, the mastoid, semicircular canal, cerebellopontine angle structures and cranial nerve. It is synonymous with Heffner tumor, low grade adenocarcinoma of endolymphatic sac origin, and aggressive papillary middle ear tumor according to the recently published World Health Organization tumor classification. These tumors can be encountered sporadically or in Von Hippel-Lindau (VHL) disease. Because of the rarity of this tumor, it can easily be confused with other tumors such as paraganglioma, middle ear adenoma, adenocarcinoma, papillary carcinoma of thyroid or choroid plexus papilloma. This report presents a 31-year-old male patient with endolymphatic sac tumor who was initially diagnosed with paraganglioma, with the following objectives: 1) to improve understanding of the variable clinical presentation of endolymphatic sac tumor, and 2) to identify histomorphological and immunohistochemical features of endolymphatic sac tumor.

## Case presentation

### Clinic history

**Figure 1:**
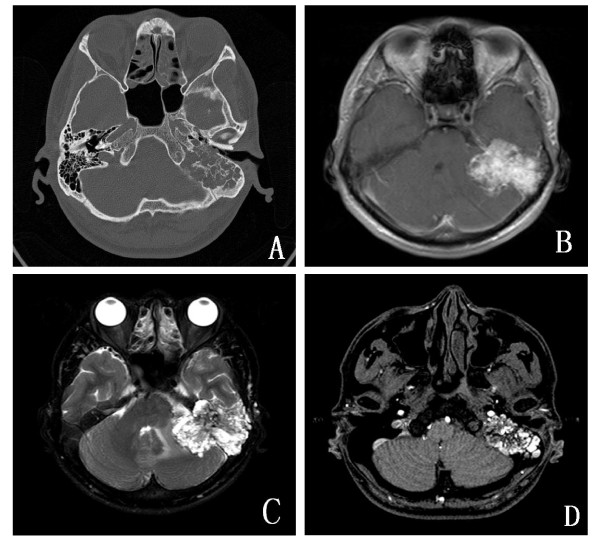
**Radiologic characterization of endolymphatic sac tumor**. **A**. CT imaging demonstrated an expansile lytic lesion of the mastoid process of the left petrous bone extending to involve both the left medial mastoid as well as the middle ear. **B**. MR scan showed a 5.2 cm × 4.7 cm × 4.2 cm mass which was irregular, heterogeneous, and lobulated and showed hyperintensity on T1-weighted images. **C**. The periphery of the lesion appeared markedly hyperintense on the T2-weighted images. **D**. Axial postcontrast T1-weighted MR scan showed a heterogeneous enhancing mass.

The patient presented with a chief complaints of progressive hearing loss of approximately 4-year duration. In the past month, the symptom increased accompanied by headaches. There was no tinnitus, otalgia, otorrhea, vertigo or facial nerve paralysis. There was no history of trauma or surgeries. On physical examination, the hearing of left ear disappeared. The right ear was normal. Examination of the facial nerves, nasopharynx, oral cavity, larynx and neck was normal. Neither the symptoms nor a family history of VHL disease were found in the patient. Computed tomography (CT) demonstrated a large, hypodense, and expansile lytic lesion of the mastoid process of the left petrous bone extending to involve both the left medial mastoid as well as the middle ear (Figure 1A). Magnetic resonance (MR) revealed a 5.2 cm × 4.7 cm × 4.2 cm mass in the mastoid process of the left petrous bone which was irregular, heterogeneous, and lobulated and showed hyperintensity on T1- and T2-weighted images (Figure 1B). The periphery of the lesion appeared markedly hyperintense on the T2-weighted images (Figure 1C). This might represent hemorrhage (extracellular methemoglobin) or cystic areas with high protein within the lesion. Axial postcontrast T1-weighted MR scan showed a heterogeneous enhancing mass (Figure 1D). Surgical excision was performed. Because the mass was too large, it was difficult to extirpate surgically. After surgery, the patient underwent gamma-knife radiosurgery for residual tumor.

## Materials and methods

Formalin-fixed, paraffin-embedded tissues from the ELST was available. These were routinely processed and stained with hematoxylin and eosin (H&E). In addition to H&E, selected sections were stained with periodic acid-Schiff (PAS).

An immunohistochemical study was performed by EnVision method. The antibodies used were cytokeratin (Pan), epithelial membrane antigen (EMA), cytokeratin 19, cytokeratin 5/6, cytokeratin 7, vimentin, CD56, glial fibrillary acidic protein (GFAP), NSE, VEGF, S-100, CgA, Syn, TTF-1, thyroglobulin, calretinin and Ki-67 (DakoCytomation, Glostrup, Denmark). For antigen retrieval, slides were boiled with citrate buffer (0.01 mol/L, pH6.0) under high pressure for all the antigens. Appropriate positive and negative control experiments were performed.

### Pathological findings

Grossly, the specimen was received as an aggregate of pale pink fragments that were noted to be moderate in consistency. It was 5 cm × 4 cm × 2 cm in size.

**Figure 2:**
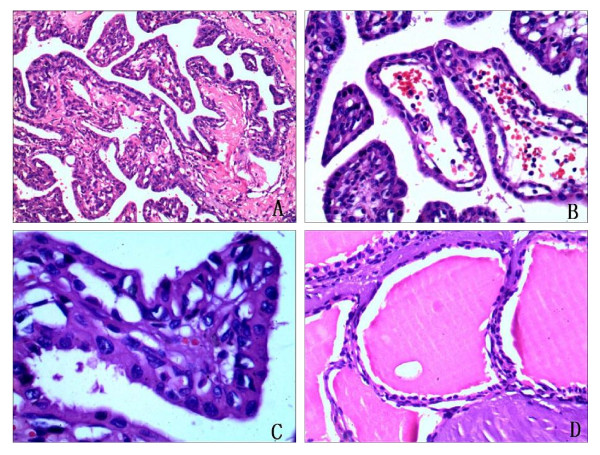
**Histopathologic characterization of endolymphatic sac tumor**. **A**. Histologic sections showed a papillary architecture (H&E, 100×). **B**. The stroma of the papillary fronds was richly vascularized(H&E, 200×). **C**. The papillary structures were lined by a single layer of flattened cuboidal-to-columnar cells. There were minimal cellular pleomorphism and rare mitotic activity (H&E, 400×). **D**. There were cystic glandular spaces filled with colloid-like material which was remarkably similar to thyroid tissue (H&E, 200×).

Morphologically, the tumor showed a papillary, cystic or glandular architecture. And the growth pattern was invasive, with destruction of petrous bone. On higher magnification the papillary and glandular structures were lined by a single layer of flattened cuboidal-to-columnar cells (Figure 2A). The stroma of the papillary fronds was richly vascularized and chronically inflamed (Figure 2B). The epithelial cells had uniform nuclei that were usually situated either in the cell center or toward the luminal aspect. They had a pale eosinophilic to clear cytoplasm (Figure 2C). Cell borders may be seen, but neoplastic cells may lack a distinct cell membrane. There were minimal cellular pleomorphism and rare mitotic activity and necrosis was not observed. There were cystic glandular spaces filled with colloid-like material which was remarkably similar to thyroid tissue (Figure 2D).

### Immunohistochemical findings

**Figure 3:**
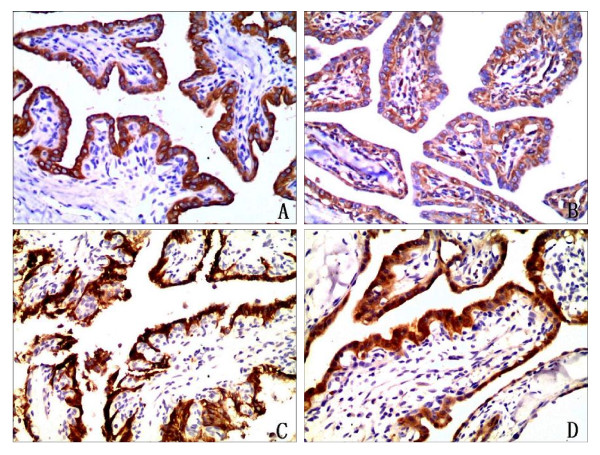
**Immunophenotype of endolymphatic sac tumor**. **A. **The tumor cells showed positive reactivity with cytokeratin (Pan) (immunostaining, 400×). **B**. The tumor cells were positive to vimentin(immunostaining, 400×). **C**. The tumor cells exhibited CD56 expression(immunostaining, 400×).**D**. The tumor cells exhibited NSE expression (immunostaining, 400×).

The tumor showed diffusely positive reactivity with cytokeratin (Pan) (Figure 3A), cytokeratin 19, cytokeratin 5/6, cytokeratin 7, EMA, vimentin (Figure 3B), CD56 (Figure 3C), and NSE (Figure 3D) and also showed variable reactivity with GFAP and VEGF. Thyroglobulin, TTF-1, S-100, and calretinin immunoreactivity was not observed. The Ki-67 immunostain showed a proliferation index of < 1%. The colloid-like material showed positive reactivity with PAS.

## Discussion

Endolymphatic sac tumor is a primary neoplasm of the temporal bone which is described in patients as young as 4 years and as old as 85 years [1-8]. Mean age of patients without VHL disease was 52.5 years, whereas in 46 patients with VHL disease the mean age was 31.3 years. Patients with VHL disease were more likely to be female (female-to male ratio of 2:1 for VHL disease patients versus 1:1 for non- VHL disease patients) [2]. Patients characteristically present with unilateral sensorineural hearing loss, tinnitus, otalgia, otorrhea, vertigo, ataxia, and facial nerve paresis. Meniere syndrome is uncommon. Large tumors growing along the posteromedial vector may cause symptoms secondary to cerebellopontine angle invasion. An indolent clinical course and long-standing symptom history are typical. The imaging hallmark of ELST is the presence of a retrolabyrinthine mass associated with osseous erosion. CT imaging demonstrated an expansile lytic lesion of the mastoid process of the petrous bone which extended to involve both the posterior fossa as well as the middle ear. In some case, the core of the tumor appeared isointense with brain on both the T1- and T2-weighted images with slight enhancement after intravenous gadolinium [9]. Our case showed hyperintensity on T1- and T2-weighted images and axial postcontrast T1-weighted MR scan showed a heterogeneous enhancing mass. The periphery of the lesion appeared markedly hyperintense on the T2-weighted images. This might represent hemorrhage (extracellular methemoglobin) or cystic areas with high protein within the lesion.

Endolymphatic sac tumors are known to occur more frequently in patients with VHL disease, with up to 16 percent of patients with VHL disease harboring an ELST [10] Patients with VHL disease are more likely to have bilateral ELSTs. VHL disease is an autosomal dominant syndrome due to deletions or mutations in the VHL gene located on short arm of chromosome 3(3p25-p26) [11]. The normal VHL gene is a tumor suppressor gene and it seems likely that the diminution of tumor suppression activity which results from the mutation of that gene leads to the various manifestations of the disease: renal cysts and renal carcinoma, pheochromocytoma, pancreatic cysts, neuroendocrine tumors, cystadenomas of the reproductive adnexal organs, and hemangioblastomas of the cerebellum, spinal cord, brain stem, and retina. VHL gene mutations have been shown both in ELSTs associated with VHL disease and in sporadic cases. Gene analysis performed to a patient with ELST who presented with a medical and family history of VHL disease showed mutation in VHL gene, resulting in C to T exchange at position 194, the consequence of which is an amino acid exchange S65L [3]. Sporadic cases of endolymphatic sac tumor without VHL disease also showed VHL gene mutation similar to that in the germ line of patients with the disease according to Hamazaki's study [12].

The origin of endolymphatic sac tumors remains controversial for many years. Several reports mentioned the mastoid or middle ear epithelium as origin of these tumors [4,13,14]. Only recently, convincing anatomic, morphological and immunohistochemical arguments exist for an endolymphatic sac origin (inner-ear origin). The tumor was first identified during sac decompression surgery in 1984 [15]. In 1989 Heffner described a new pathological entity of the endolymphatic sac. This tumor grew slowly, destroyed the temporal bone, and showed papillary cystic and glandular patterns. He also found the epicenters of the tumors in his cases and in other reports to have the same anatomic location, in the area of the vestibular aqueduct and endolymphatic sac of the posterior petrous bone. In addition, there are some common histologic features between these tumors and the normal endolymphatic sac. So he speculated that the tumor arise in the endolymphatic sac [16]. In 2004 Lonser described a 40-year-old man with von Hippel Lindau disease showing bilateral endolymphatic sac tumors. On the right side there was an extensive endolymphatic sac tumor invading the temporal bone to involve structures as far distant as mastoid air cells. On the left side illustrations define a papillary tumor confined to the lumen of endolymphatic ducts and sacs (EDS), extending from the endolymphatic duct to the extraosseous sac on the dural surface. This case described here confirms the concept of an origin of the neoplasm in the endolymphatic sac [17]. The endolymphatic duct arises in the posteroinferior part of the vestibule of the inner ear as the result of the union of an endolymph-containing epithelial tube from the saccule and one from the utricle. This tube passes into a bony canal, the vestibular aqueduct, and extends posteriorly and laterally. It soon widens in a superior-inferior plane but not in an anteroposterior one, and is here known as the endolymphatic sac. A study of the epithelium of normal human mature endolymphatic ducts and sacs (EDSs) in archival temporal bone sections showed hyperplastic tubular outgrowths, usually situated in the intraosseous portion of the endolymphatic sac, in most cases. So the intraosseous portion of the endolymphatic sac maybe the the origin of endolymhoatic sac tumor [18]. The endolymphatic sac is derived from neuroectoderm and is located subjacent to the posteromedial surface of the temporal bone. There are 4 potential vectors for endolymphatic sac tumor extension: posteromedially into the cerebellopontine angle, laterally toward the middle ear, superiorly toward the middle cranial fossa, and anteromedially along the petrous ridge toward the cavernous and sphenoid sinuses. Individual examples may grow primarily along one of these vectors or along multiple vectors [19].

Histopathologically, these tumors are composed of papillary and cystic proliferations lined by a single layer of flattened cuboidal-to-columnar cells. The nuclei of the epithelial layer show slight variability in size and shape. Mitotic activity is rare. The stroma of the papillary fronds was richly vascularized and chronically inflamed. Large areas of hypocellular fibrosis, hemorrhage, cholesterol clefts, and associated reactive changes are frequently found. Because of the rarity of this tumor, that can easily be confused with other papillary lesions on histopathologic grounds. The histopathological differential diagnosises of ELST include paraganglioma, chorioid plexus papilloma, papillary ependymoma, papillary mesohyloma, middle ear adenoma, and metastatic carcinoma of the thyroid, kidney, prostate, lung, and breast. Paragangliomas of the middle ear are cytologically benign and may demonstrate papillary structures and cuboidal cells with eosinophilic cytoplasm. However, paraganglioma cells are positive for chromogranin and synaptophysin, and at least focally they are arranged in characteristic nests. This tumor we report was positive for cytokeratin (Pan), cytokeratin 19, cytokeratin 5/6, cytokeratin 7, and epithelial membrane antigen, so it is easy to differentiate them by immunohistochemistry. Chorioid plexus papilloma may be histologically similar to ELST, but it originates in the ventricle and does not invade bone. To aid in the differentiation between papillary tumors of endolymphatic sac and duct origin and those arising from ectopic choroid plexus, an immunohistochemical study using stains for transthyretin (TTR), cytokeratins, S-100 protein, EMA, and GFAP was carried out on archival specimens of normal and neoplastic endolymphatic sac and duct and choroid plexus epithelium. Transthyretin, a marker for choroid plexus epithelium, was found to show differential expression between choroid plexus papillomas and aggressive papillary tumors of the endolymphatic sac or duct. Therefore the use of TTR in concert with other immunohistochemical stains appear to aid in the differentiation between intracranial and intratemporal papillary tumors arising from choroid plexus and endolymphatic sac or duct epithelium [20]. Papillary ependymoma shows discohesive growth, pseudopapillae, and perivascular pseudorosettes. Finger like projections are lined by single or multiple layers of cuboidal tumor cells with smooth contiguous surfaces and with GFAP-positive tumor cell processes. Papillary mesohyloma occurs in pelvic peritoneum, gastrocolic omentum, mesenterium, pleura and is positive to CK5/6, calretinin, and vimentin. Middle ear adenomas are nonpapillary, are confined to the middle ear and mastoid, and rarely show bone destruction. Currently, considerations of metastases from thyroid, breast, and prostate can be eliminated by immunohistochemistry.

Treatment of ELST involves local resection of the tumor or radical mastoidectomy alone, or in combination with radiotherapy. Early surgical excision is the best treatment when the tumor is small. Remission may last for years. When the tumor is large, complete excision is nearly impossible due to the anatomic complexity of the endolymphatic sac and distinct patterns of extension. Local recurrence after surgery can occur. In advanced cases radiotherapy alone may be the sole option, although there are no studies focusing on the role of adjuvant radiotherapy. Because endolymphatic sac tumor is richly vascularized, some scholars suggest that preoperative embolization may be helpful. In our case, the mass was large, destructed the mastoid process of the left petrous bone and extended to involve both the left medial mastoid as well as the middle ear, so it was difficult to extirpate surgically. After surgery, the patient underwent gamma-knife radiosurgery for residual tumor.

## Consent

Written informed consent was obtained from the patient for publication of this case report and any accompanying images. A copy of the written consent is available for review by the Editor-in-Chief of this journal.

## Competing interests

The authors declare that they have no competing interests.

## Authors' contributions

Y-HS constructed the manuscript and carried out pathological examination. K-XW was responsible for the clinical data. WW, J-HW and HG participated in pathological investigations. M-QX carried out the immunohistochemistry. J-MS revised manuscript critically for important intellectual content and had given final approval of the version to be published. All authors read and approved the final manuscript.
